# Replication of recently identified systemic lupus erythematosus genetic associations: a case–control study

**DOI:** 10.1186/ar2698

**Published:** 2009-05-14

**Authors:** Marian Suarez-Gestal, Manuel Calaza, Emöke Endreffy, Rudolf Pullmann, Josep Ordi-Ros, Gian Domenico Sebastiani, Sarka Ruzickova, Maria Jose Santos, Chryssa Papasteriades, Maurizio Marchini, Fotini N Skopouli, Ana Suarez, Francisco J Blanco, Sandra D'Alfonso, Marc Bijl, Patricia Carreira, Torsten Witte, Sergio Migliaresi, Juan J Gomez-Reino, Antonio Gonzalez

**Affiliations:** 1 Laboratorio de Investigacion 10 and Rheumatology Unit, Hospital Clinico Universitario de Santiago, Santiago de Compostela 15706, Spain; 2 Paediatrics Department, Albert Szent-Györgyi Medical and Pharmaceutical Centre, University of Szeged, Szeged 6721, Hungary; 3 Institute of Clinical Biochemistry, Martin Faculty Hospital, Jessenius Medical Faculty, Kollárova 2, 036 59 Martin, Slovakia; 4 Internal Medicine, Research Laboratory in Autoimmune Diseases, Hospital Vall d'Hebron, 08035 Barcelona, Spain; 5 Ospedale S Camillo-Forlanini, U O Complessa di Reumatologia, 00151 Roma, Italy; 6 Molecular Biology and Immunogenetics Department, Institute of Rheumatology, 128 50 Prague 2, Czech Republic; 7 Rheumatology Department, Hospital Garcia de Orta, Almada, Portugal; 8 Rheumatology Research Unit, Instituto Medicina Molecular, Faculdade de Medicina da Universidade de Lisboa, Portugal; 9 Department of Histocompatibility and Immunology, Evangelismos Hospital, 10676 Athens, Greece; 10 Clinical Immunology, University of Milan and Fondazione IRCCS Ospedale Maggiore Policlinico, Mangiagalli e Regina Elena, 20122 Milan, Italy; 11 Pathophysiology Department, Athens University Medical School, Athens 115 27, Greece; 12 Department of Functional Biology, Hospital Universitario Central de Asturias, Universidad de Oviedo, Oviedo 33006, Spain; 13 INIBIC-CH Universitario A Coruña, 15006 A Coruña, Spain; 14 Dept Medical Sciences and IRCAD, Eastern Piedmont University, 28100 Novara, Italy; 15 Department of Rheumatology and Clinical Immunology, University Medical Center Groningen, 9713 Groningen, The Netherlands; 16 Rheumatology Unit Hospital 12 de Octubre, 28041 Madrid, Spain; 17 Division of Clinical Immunology, Department of Internal Medicine of the Hannover Medical School, D-30625 Hannover, Germany; 18 Rheumatology Unit, Second University of Naples, 81100 Naples, Italy; 19 Department of Medicine, University of Santiago de Compostela, Santiago de Compostela, 15706, Spain

## Abstract

**Introduction:**

We aimed to replicate association of newly identified systemic lupus erythematosus (SLE) loci.

**Methods:**

We selected the most associated SNP in 10 SLE loci. These 10 SNPs were analysed in 1,579 patients with SLE and 1,726 controls of European origin by single-base extension. Comparison of allele frequencies between cases and controls was done with the Mantel–Haenszel approach to account for heterogeneity between sample collections.

**Results:**

A previously controversial association with a SNP in the *TYK2 *gene was replicated (odds ratio (OR) = 0.79, *P *= 2.5 × 10-^5^), as well as association with the X chromosome *MECP2 *gene (OR = 1.26, *P *= 0.00085 in women), which had only been reported in a single study, and association with four other loci, *1q25.1 *(OR = 0.81, *P *= 0.0001), *PXK *(OR = 1.19, *P *= 0.0038), *BANK1 *(OR = 0.83, *P *= 0.006) and *KIAA1542 *(OR = 0.84, *P *= 0.001), which have been identified in a genome-wide association study, but not found in any other study. All these replications showed the same disease-associated allele as originally reported. No association was found with the *LY9 *SNP, which had been reported in a single study.

**Conclusions:**

Our results confirm nine SLE loci. For six of them, *TYK2*, *MECP2*, *1q25.1*, *PXK*, *BANK1 *and *KIAA1542*, this replication is important. The other three loci, *ITGAM*, *STAT4 *and *C8orf13-BLK*, were already clearly confirmed. Our results also suggest that *MECP2 *association has no influence in the sex bias of SLE, contrary to what has been proposed. In addition, none of the other associations seems important in this respect.

## Introduction

Systemic lupus erythematosus (SLE) is a complex autoimmune disease of wide variability in its manifestations and clinical evolution that characteristically involves multiple autoantibodies against ubiquitous nuclear antigens. Its genetic component is very significant, as shown by a sibling recurrence risk ratio of 20 and a 10-fold excess in SLE concordance between monozygotic twins over dizygotic twins [1,2].

Linkage studies have indicated that this genetic component is due to multiple low-penetrance common genetic factors [1]. Only a few factors had consistently been demonstrated until 2008: the class II HLA alleles, low-affinity receptors for the constant fraction of IgG, and the *PTPN22 *and *IRF5 *genes. This scenario has been dramatically improved by new technologies and genome resources [2]. Four genome-wide association (GWA) studies were published in 2008 [3-6] that, together with other large-scale studies, have greatly enlarged the number of convincing SLE-associated loci. Not all of the newly described findings, however, have attained the same degree of confirmation [2]. Some of them are already definitively confirmed by replication in different sample collections by the same authors and also by independent authors in separate studies (Table 1). In this group are the SLE associations with the *ITGAM *[3,4,6,7], *STAT4 *[3,4,6,8-12] and *C8orf13-BLK *regions [3,4,6]. Other findings are very solid but they still require confirmation by independent studies. In this group are the associated loci that were only reported in a single GWA study but not in the other studies, such as *BANK1 *[5], *PXK *[3], *KIAA1542 *[3] and *1q25.1 *[3], or those that were reported in a single large study but not in any of the four GWA studies, such as *MECP2 *[13] and *LY9 *[14]. Finally, the *TYK2 *association is more controversial because it was found in a large study with Scandinavian families [15], partially replicated in a large study of UK families [16], and excluded in one of the GWA studies [3].

**Table 1:** Newly systemic lupus erythematosus-associated loci that were examined with previous evidence of association

Locus	Chr.	Location	SNP	Alleles^a^	OR (95% CI)	Associated	Sample size	Population	Reference
*ITGAM*	16	Exon 3	rs1143679	G/A	1.78 (1.6 to 2.0)	Yes	3,818	European American	[7]
					1.55 (1.2 to 2.0)	Yes	1,289	African American	[7]
					2.07 (1.3 to 3.4)	Yes	271	Gullah	[7]
			Other SNPs			Yes			[3,4,6]
*STAT4*	2	Intron 3	rs7574865	G/T	1.5 (1.2 to 1.8)	Yes	3,057	European	[3]
					1.50 (1.4 to 1.7)	Yes	4,651	European American	[4]
					1.55 (1.3 to 1.8)	Yes	2,287	European American	[8]
					1.61 (1.4 to 1.9)	Yes	2,495	Japanese	[9]
					1.62 (1.2 to 2.2)	Yes	565	Colombians	[10]
					1.56 (1.4 to 1.7)	Yes	3,958	European	[12]
			Other SNPs			Yes			[6,11]
*C8orf13-BLK*	8	Intergenic	rs13277113	G/A	1.39 (1.3 to 1.5)	Yes	6,301	European American	[4]
			Other SNPs			Yes			[3,6]
*TYK2*	19	Exon 8	rs2304256	C/A	0.625 (0.5 to 0.8)	Yes	966	Swedish/Finnish	[15]
					105:127^b^	No	380^c^	European/Indo-Pakistani	[16]
			Other SNPs			No			[3]
*MECP2*	X	Intron 2	rs17435	A/T	1.58 (1.3 to 1.9)	Yes	1,364	Korean	[13]
					1.29 (1.1 to 1.5)	Yes	2,160	European	[13]
*1q25.1*	1	Intergenic	rs10798269	G/A	0.82 (0.8 to 0.9)	Yes	6,728	European	[3]
*BANK1*	4	Intron 1	rs17266594	T/C	0.74 (0.6 to 0.9)	Yes	927	Scandinavian	[5]
					0.70 (0.5 to 0.9)	Yes	576	German	[5]
					0.63 (0.5 to 0.8)	Yes	450	Italian	[5]
					0.78 (0.6 to 0.9)	Yes	1,136	Spanish	[5]
					0.58 (0.4 to 0.8)	Yes	620	Argentinian	[5]
*KIAA1542*	11	Intron 4	rs4963128	G/A	0.78 (0.7 to 0.9)	Yes	6,728	European	[3]
*PXK*	3	Intron 5	rs6445975	T/G	1.25 (1.2 to 1.4)	Yes	6,728	European	[3]
*LY9*	1	Exon 8	rs509749	A/G	377:403^d^	Yes	510^c^	UK Caucasian	[14]
					237:251^d^	No	270^c^	Canadian	[14]

Loci ordered as presented in Table 2. The SNPs selected for replication are detailed. Chr, chromosome; CI, confidence interval; OR, odds ratio. ^a^Major/minor alleles. ^b^Transmitted:untransmitted. ^c^Number of families. ^d^Observed:expected.

In the present paper, therefore, we have analysed SLE association to each of these loci in more than 1,500 SLE patients and 1,700 controls – and all of them except *LY9 *have been clearly replicated. In addition, we have found that many of these loci are also important for SLE in men where data from previous reports is almost completely absent.

## Materials and methods

### Sample collection

We used DNA samples from SLE patients and ethnically matched healthy controls of 16 collections from nine European countries (see Table S1 in Additional data file 1). Most of these samples have already been described [17]. Two new sample collections were from Asturias, Spain and Almada, Portugal. Each recruiting centre was asked for about 100 SLE patients and 100 ethnically matched controls. A total of 1,579 cases and 1,726 controls were obtained in this way. All SLE patients met the revised American College of Rheumatology classification criteria [18]. Clinical characteristics of the patients are provided in Table S2 in Additional data file 1. Patients and controls gave written informed consent. Sample collection was approved by the respective ethical committees.

### Genotyping

We selected a SNP for each of the 10 associated loci that we intended to replicate (Table 1). The SNPs were selected because they were strongly associated with SLE or because they were described as probable causal polymorphisms. These 10 SNPs were amplified in a single PCR with the Qiagen Multiplex PCR kit (Qiagen, Chatsworth, CA, USA) with 20 ng genomic DNA and 0.2 μM of each primer (for primers and probes, see Table S3 in Additional data file 1). The PCR products were purified by digestion with Exonuclease I (Epicentre, Madison, WI, USA) and shrimp alkaline phosphatase (GE Healthcare, Barcelona, Spain). Purified PCR products were genotyped by single-base extension with the SNaPshot Multiplex Kit (Applied Biosystems, Foster City, CA, USA) and specific probes. After a second purification with shrimp alkaline phosphatase (GE Healthcare), samples were analysed in the Abi Prism 3130xl Genetic Analyzer (Applied Biosystems) and genotypes assigned by the GeneMapper software. All genotype calls were manually reviewed and conflicting results were liberally re-assayed or re-genotyped by sequencing with the Big Dye Ready Reaction Kit v 3.1 (Applied Biosystems). Sequence reactions followed the kit manufacturer protocol and were also analysed in the Abi Prism 3130xl Genetic Analyzer.

### Statistical analysis

Some of the sample collections in our study have already been used for the analysis of specific associations included in this project. They have been excluded from the relevant analyses to avoid data duplication; this circumstance is detailed in Table S4 in Additional data file 1, where raw genotype data from each sample collection are reported. Hardy-Weinberg equilibrium tests in control samples were performed with Haploview with a threshold of 0.05 uncorrected for multiple tests [19]. Other statistical analyses were carried out in a customized version of the Statistica 7.0 program (StatSoft, Tulsa, OK, USA).

Comparison of cases and controls was carried out with the Mantel–Haenszel approach because allele frequency differences are probable between sample collections even if specific effects on the phenotype are constant. Spurious false positive or false negative results therefore become likely if the allele differences are not accounted for. To avoid this, the Mantel–Haenszel approach combines effect sizes taken as the odds ratio (OR) in each stratum allowing for heterogeneity in allele frequencies. This approach provides an accurate combined statistic if the heterogeneity of effect sizes, evaluated with the Breslow–Day test, is excluded. Significant heterogeneity of effects is therefore excluded by the Breslow–Day test and allele frequency heterogeneity is accounted for with the Mantel–Haenszel approach. These analyses were also conducted after stratifying the samples by gender. Univariate logistic regression models were used to test the fit to the data of additive, recessive and dominant genetic models. Statistical power was estimated with the Power and sample size calculations software [20].

## Results

A total of 1,579 SLE patients and 1,726 controls from 16 European collections were available for study (Tables S1 and S2 in Additional data file 1). The genotyping call rate was 99.9% and the genotypes in controls were in Hardy-Weinberg equilibrium. Individual collection data for each SNP is shown in Tables S4 and S5 in Additional data file 1. Combined analysis of the SNP effects across our sample collections was performed with the Mantel–Haenszel approach, which is a method correcting for variability in allele frequencies between collections provided that the effect sizes (that is, ORs) are not significantly divergent. This condition was fulfilled because no significant heterogeneity in OR was detected for any of the SNPs (Table 2, final column).

**Table 2:** Combined analysis of allele frequency differences between SLE cases and controls for nine autosomal loci

	Minor allele frequency (%)^a^		Mantel – Haenszel analysis		Breslow – Day test
			
SNP (locus)	SLE cases	Controls	OR (95% CI)	*P *value	*P *value
rs1143679 (*ITGAM*)	23.2 (730/3,152)	15.1 (521/3,448)	1.70 (1.5 to 1.9)	1.1 × 10^-16^	0.5
rs7574865 (*STAT4*)	32.8 (709/2,162)	23.2 (485/2,092)	1.62 (1.4 to 1.9)	2.4 × 10^-12^	1.0
rs13277113 (*C8orf13-BLK*)	30.9 (874/2,824)	25.7 (776/3,024)	1.34 (1.2 to 1.5)	5.1 × 10^-7^	1.0
rs2304256 (*TYK2*)	23.3 (733/3,152)	27.8 (960/3,450)	0.79 (0.7 to 0.9)	2.5 × 10^-5^	0.6
rs10798269 (*1q25.1*)	27.3 (861/3,158)	31.8 (1,098/3,452)	0.81 (0.7 to 0.9)	0.00013	0.2
rs17266594 (*BANK1*)	24 (526/2,192)	27.6 (624/2,260)	0.83 (0.7 to 0.9)	0.0062	0.4
rs4963128 (*KIAA1542*)	30.3 (955/3,150)	34.0 (1,173/3,448)	0.84 (0.8 to 0.9)	0.0011	0.2
rs6445975 (*PXK*)	27.3 (772/2,824)	24.2 (734/3,034)	1.19 (1.1 to 1.3)	0.0038	0.7
rs509749 (*LY9*)	43.1 (1,359/3,154)	43.8 (1,513/3,452)	0.97 (0.9 to 1.1)	0.5	0.4

Loci ordered by decreasing effect size (odds ratio (OR)). All results refer to the minor allele of each SNP, which is indicated in Table 1. CI, confidence interval; SLE, systemic lupus erythematosus. ^a^Data presented as percentage (number of minor alleles/total number of alleles).

The combined data showed significant differences between SLE cases and controls for eight of the nine SNPs located in autosomal chromosomes (Table 2). All of the significant differences between cases and controls were in the same direction as originally reported (Tables 1 and 2). We found association of the four SNPs that have been reported in a single GWA and not yet replicated by independent studies: rs10798269 in *1q25.1 *(OR = 0.81, *P *= 0.00013), rs6445975 in *PXK *(OR = 1.19, *P *= 0.0038), rs17266594 in *BANK1 *(OR = 0.83, *P *= 0.0062) and rs4963128 in *KIAA1542 *(OR = 0.84, *P *= 0.0011). There was also significant association of two of the three SNPs that were described in large studies but that were not observed in any of the GWA studies: rs2304256 in *TYK2 *(OR = 0.79, *P *= 2.5 × 10-^5^) and rs17435 in *MECP2 *(analysis of this SNP was performed separately in women and men because this gene is in chromosome X; see below). Only rs509749 in *LY9 *was similar in cases and controls. Our study had sufficient power (80%) to detect association at this SNP with an effect size equivalent to OR > 1.15 with *P *< 0.05 (or OR > 1.23 for *P *< 0.001).

In addition to these important results for replication, we found association with the three loci that have already been replicated in GWA studies: rs1143679 in *ITGAM *(OR = 1.70, *P *= 1.1 × 10-^16^), rs7574865 in *STAT4 *(OR = 1.62, *P *= 2.4 × 10-^12^) and rs13277113 in *C8orf13-BLK *(OR = 1.34, *P *= 5.1 × 10^-7^). The effect sizes of these three association signals (that is, their ORs) were larger than for all the other signals, perhaps explaining the more consistent replication of their association. Genotype comparisons for the different SNPs were concordant with an additive genetic model and yielded very similar results to the allele frequency analyses (data not shown).

Combined analysis was also conducted in women (Table 3). This was particularly necessary for the *MECP2 *SNP rs17435, located in the X chromosome. This SNP showed a significant difference between SLE women and control women and with the same disease-associated allele as previously reported (OR = 1.26, *P *= 0.00085). The SNPs placed in the autosomes showed similar results to those obtained in the unstratified analysis. There were only less significant *P *values due to the smaller sample size, but the effect sizes (expressed as ORs) remained largely unchanged. The *BANK1 *SNP was not associated in women, but this was the SNP with fewer available samples because we have excluded from this analysis the sample collections that have previously been reported (power was 0.68 for *P *= 0.05 and OR = 0.78, which was previously reported in Spanish samples) [5].

**Table 3:** Combined analysis of allele frequency differences between SLE women and control women

	Minor allele frequency (%)^a^		Mantel – Haenszel analysis		Breslow – Day test
			
SNP (locus)	SLE cases	Controls	OR (95% CI)	*P *value	*P *value
rs1143679 (*ITGAM*)	22.3 (621/2,782)	15.1 (329/2,182)	1.67 (1.4 to 2.0)	2.0 × 10^-11^	0.6
rs7574865 (*STAT4*)	33.1 (636/1,920)	24.0 (317/1,322)	1.60 (1.4 to 1.9)	8.4 × 10^-9^	0.8
rs13277113 (*C8orf13-BLK*)	30.9 (777/2,514)	25.7 (475/1,848)	1.33 (1.2 to 1.5)	5.4 × 10^-5^	0.9
rs2304256 (*TYK2*)	22.9 (638/2,782)	27.2 (592/2,180)	0.81 (0.7 to 0.9)	0.0022	0.2
rs17435 (*MECP2*)	26.7 (744/2,784)	22.8 (498/2,186)	1.26 (1.1 to 1.4)	0.00085	0.7
rs10798269 (*1q25.1*)	27.1 (754/2,786)	32.3 (705/2,182)	0.77 (0.7 to 0.9)	6.2 × 10^-5^	0.4
rs17266594 (*BANK1*)	23.9 (464/1,938)	26.9 (386/1,436)	0.87 (0.7 to 1.0)	0.077	0.4
rs4963128 (*KIAA1542*)	30.4 (845/2,778)	33.9 (741/2,182)	0.85 (0.8 to 1.0)	0.011	0.2
rs6445975 (*PXK*)	26.7 (670/2,514)	23.5 (436/1,854)	1.22 (1.1 to 1.4)	0.0067	0.9
rs509749 (*LY9*)	43.3 (1,206/2,784)	44.0 (962/2,184)	0.98 (0.9 to 1.1)	0.7	0.5

Loci ordered as presented in Table 2. All results refer to the minor allele of each SNP, which is indicated in Table 1. CI, confidence interval; OR, odds ratio; SLE, systemic lupus erythematosus. ^a^Data presented as percentage (number of minor alleles/total number of alleles).

No previous detailed information of men with SLE has been published for any of these associated loci, although in a report describing association of the *ITGAM *gene it was indicated that results were not different between women and men [7]. This lack of information is probably due to the rarity of men suffering from SLE. In our analysis, we have considered all male data together without stratifying for sample collection due to the low number of men in each collection (Table 4). Results in men were similar to results in women, with the possible exception of the rs1143679 in *ITGAM *(OR = 2.08 versus 1.67; *P *= 0.03). Some SNPs were not associated in men (in the *TYK2*, *1q25.1*, *BANK1 *and *LY9 *loci), but statistical power of this subgroup analysis was low, ranging from 0.19 for rs17266594 in *BANK1 *to 0.25 for rs2304256 in *TYK2 *among the nonassociated SNPs (power was estimated for *P *= 0.05 and OR = 1.2).

**Table 4:** Comparison of SNP allele frequencies between SLE men and control men

	Minor allele frequency (%)^a^		Mantel – Haenszel analysis	
		
SNP (locus)	SLE cases	Controls	OR (95% CI)	*P *value
rs1143679 (*ITGAM*)	27.9 (83/298)	15.7 (181/1,154)	2.08 (1.5 to 2.8)	1.2 × 10^-6^
rs7574865 (*STAT4*)	31.7 (64/202)	21.5 (159/740)	1.69 (1.2 to 2.4)	0.0025
rs13277113 (*C8orf13-BLK*)	31.0 (88/284)	25.1 (267/1,064)	1.34 (1.0 to 1.8)	0.045
rs2304256 (*TYK2*)	26.8 (80/298)	28.8 (334/1,158)	0.91 (0.7 to 1.2)	0.5
rs17435 (*MECP2*)^b^	29.2 (42/144)	18.5 (105/568)	1.82 (1.2 to 2.8)	0.0046
rs10798269 (*1q25.1*)	28.0 (84/300)	30.7 (355/1,158)	0.88 (0.7 to 1.2)	0.4
rs17266594 (*BANK1*)	26.2 (56/214)	28.4 (227/798)	0.89 (0.6 to 1.3)	0.5
rs4963128 (*KIAA1542*)	28.0 (84/300)	34.9 (403/1,156)	0.73 (0.6 to 1.0)	0.025
rs6445975 (*PXK*)	32.1 (86/268)	24.8 (265/1,068)	1.43 (1.1 to 1.9)	0.016
rs509749 (*LY9*)	40.6 (121/298)	42.9 (496/1,156)	0.91 (0.7 to 1.2)	0.5

Loci ordered as presented in Table 2. All results refer to the minor allele of each SNP, which is indicated in Table 1. No stratified analysis by sample collection was done due to the small number of patients with systemic lupus erythematosus (SLE) in each collection. CI, confidence interval; OR, odds ratio. ^a^Data presented as percentage (number of minor alleles/total number of alleles). ^b^These results are of carrier analysis because *MECP2 *is in the X chromosome and there is a single allele in each man.

## Discussion

Our aim has been to contribute to the definition of consistent SLE genetic factors derived from recent sound studies: four of the associations have been described in a GWA study, but not in a second GWA study or any other study; another two associations were identified in large studies, but not in any of the GWA studies; and one association is more controversial (Table 1). Our results are highly reassuring because all of the associations, except one from the group not found in any GWA study, were replicated with clarity and showed the same disease-associated allele as originally reported. This high degree of reproducibility is a fundamental change that large studies have brought to genetic research of SLE and other complex diseases [2,21]. This change allows a bright future for the investigation of the genetic component of SLE.

The most remarkable result from the present study has probably been the association signal observed with the rs2304256 nonsynonymous SNP of *TYK2 *(OR = 0.79) because this has been a controversial SLE genetic factor. The rs2304256 SNP introduces a valine to phenylalanine change in the Janus homology domain 4 of *TYK2 *whose functional relevance has not yet been tested. This nonsynonymous SNP showed the strongest association among the 11 *TYK2 *SNPs studied in Scandinavian families [15], but was not associated in a study of UK families [16]. This latter study, however, found association with another *TYK2 *SNP (rs12720270) that was not associated in the Scandinavian study. Finally, the International Consortium for Systemic Lupus Erythematosus Genetics (SLEGEN) GWA study excluded association with the rs12720270 SNP (the rs2304256 SNP was not included in the GWA panels) [3]. Our results are important in this context because they show a significant association that confirms the role of the rs2304256 nonsynonymous SNP. In addition, combined analysis of all available data show a clear SLE association (*P *= 2.10 × 10-^11^) that is stronger than the required for genome-wide significance.

Tyk2 is a Janus-family tyrosine kinase that is bound to cytokine receptors and becomes activated after ligand binding. Deficiency of *TYK2 *leads to defects of multiple cytokine pathways, including type I interferon, IL-6, IL-10, IL-12, and IL-23, and to impaired T-helper type 1 differentiation and accelerated T-helper type 2 differentiation [22]. Only future research will indicate which of these pathways is critically affected by the *TYK2 *risk allele.

Following in importance is the association of *MECP2 *because our results provide replication and indicate that a previous assumption about the role of this genetic factor in contributing to the sex bias in SLE is questionable. Sawalha and colleagues considered the X-chromosome methyl CpG binding protein 2 coding gene (*MECP2*) as a possible SLE genetic factor based on two features: SLE predominance in women and abnormal regulation of methylation-sensitive T-cell genes in SLE [13]. *MECP2 *could be involved in both phenomena because this gene is in the X chromosome and participates in DNA methylation. Sawalha and colleagues found association with several SNPs in women from two ethnic groups, Korean and European (OR for rs17435 = 1.58 and 1.29, respectively) [13]. The association we have found in women (OR = 1.26) is very similar to that reported in their European sample, providing strong confirmatory evidence. This replication is important for the status of *MECP2 *due to the lack of association signals in the SLE GWA studies.

In addition, we have found that the *MECP2 *SNP is also associated with SLE in men (OR = 1.82, *P *= 0.0046), which was not previously known. This result seriously undermines the hypothesized role of *MECP2 *in SLE gender bias. In retrospect, lack of sex specificity is congruent with experiments that showed *MECP2 *is not expressed in the inactivated X chromosome of women [23], which implies expression levels in men and women should be equivalent. Future research should aim to establish whether any of the SLE-associated SNPs in *MECP2 *has a functional effect and to find evidence of the hypothesized relationship between altered methylation of T-cell genes in SLE and *MECP2*. In addition, it is even unclear whether the causal polymorphism affects *MECP2 *because SLE association has also been reported with genetic variants in a neighbour gene, *IRAK1*, which is a key mediator in the signalling pathways of Toll-like receptors/IL-1R [24].

The rs10798269 SNP in the *1q25.1 *locus, the rs4963128 SNP in the *KIAA1542 *gene and the rs6445975 SNP in the *PXK *gene were reported in the SLEGEN GWA study [3] with *P *values below 2 × 10^-7^, but they were not reported in Hom and colleagues' GWA study [4] and none of them has yet been replicated in any other study. The three SNPs were associated with SLE in our study, with effect sizes that are similar to those reported (OR = 0.81 versus 0.82 for the *1q25.1 *SNP, 0.84 versus 0.78 for the *KIAA1542 *SNP, and 1.19 versus 1.25 for the *PXK *SNP). None of these three SNPs has any predictable functional effect. In addition, the rs10798269 SNP in the *1q25.1 *locus is far from any known transcript and the *PXK *and *KIAA1542 *genes are of unknown function. The *KIAA1542 *gene, however, is about 20 kb away from the *IRF7 *gene and in linkage disequilibrium with it, raising the possibility that this association could be related with *IRF7 *function [3]. Our replication of these associations increases the need for research aimed to the identification of their functional effects.

We have also found a significant association with the rs17266594 in the *BANK1 *gene. This SLE genetic factor has been identified in a low-resolution GWA study in a Swedish sample and replicated in other European sample collections in the same study [5], but it was not found in any of the high-resolution GWA studies and has not yet been replicated by other groups. Our results provide this independent replication, although with a more modest effect (OR = 0.83 in our study versus 0.70 in Kozyrev and colleagues [5]). The causal polymorphism can be the rs17266594 SNP itself, which seems to alter splicing efficiency of *BANK1*, or two *BANK1 *nonsynonymous SNPs of possible damaging effect. Linkage disequilibrium between these three SNPs has prevented dissection of their relationship to SLE susceptibility [5]. *BANK1 *codes for a B-cell scaffold protein with ankyrin repeats that is implicated in B-cell receptor-mediated signalling.

The rs509749 SNP of *LY9 *is the only SNP that was not replicated in our study. We selected this SNP because it seems to explain the 1q23 SLE-linked locus according to a large family-based study [14]. 1q23 is one of the most consistently described SLE loci in linkage studies (and its syntenic region in the mouse lupus models) [1]. Examination of SNPs all along this locus showed stronger association with the rs509749 SNP [14]. This SNP has a predictable impact in protein function and is associated with changes in the proportion of specific T-cell subsets [14]. All this evidence made the rs509749 SNP a good candidate for replication in our view, even if the level of significance of the SLE association was notably lower than the reported for the other nine SNPs studied here (*P *= 0.002). Lack of replication of this SNP in contrast with replication of the other nine SNPs provides support for the direct relationship between very low *P *values obtained in sound studies and the reproducibility of genetic association findings [21].

The most associated SNPs in our samples were the three that were already confirmed previous to our study. These three SNPs were associated with SLE in at least three large studies. The largest effect was observed with a nonsynonymous SNP in the third exon of the *ITGAM *gene (rs1143679, OR = 1.70) [3,4,6,7]. This nonsynonymous SNP was the most associated in one of the previous studies (with very similar effect, OR = 1.74) [7], and has been hypothesized to disturb ITGAM interaction with its ligands, but still no functional evidence is available. Another clearly established association [3,4,6,8-12] was the second strongest in our study: SNP rs7574865 in the third intron of the *STAT4 *gene (OR = 1.62). This association seems stronger in patients with a severe phenotype [12]; however, no functional polymorphism has been identified in this locus. The next strongest association (OR = 1.34) was with the rs13277113 SNP, which has been reported in the GWA study of Hom and colleagues [4], with a similar effect (OR = 1.39). This SNP is located between *C8orf13 *(of unknown function) and *BLK *(B-lymphoid tyrosine kinase), two genes that are transcribed in opposite directions. No functional variant has been identified in this locus, but the risk allele of this SNP correlates with low mRNA levels of *BLK *and high levels of *C8orf13*, raising the possibility that either of these two effects could be related with SLE. Graham and colleagues found association with a strongly linked SNP in the *BLK *gene [6], while the SLEGEN GWA study found association with an unlinked SNP in this locus, suggesting the possibility of two independent genetic factors [3].

In addition, we have found that most examined SLE-associated SNPs seem to be shared between women and men. Results are not definitive given the small number of men in the patient group. This lack of differential association is important because we do not know definitively the causes of the female preference of SLE. Lack of detailed gender analysis in previous genetic reports is regrettable because only aggregation of data from multiple studies will allow us to know whether genetic factors contribute to this sex bias.

## Conclusions

In summary, our study has provided independent replication of nine SLE-associated loci, six of them of confirmatory importance because they have not yet been independently replicated by other groups (*1q25.1*, *MECP2*, *KIAA1542*, *PXK *and *BANK1*) or because their association was controversial (*TYK2*). These results bring the number of strongly confirmed associated loci to 13. Replication in independent studies is indispensable for considering a genetic factor in this category, although the common use of multiple case–control sets inside the same study or of large sample collections has increased the chances of replication [2]. Some other promising associations have been discovered [6,25], or await sufficient independent replication [2], but it is already certain that the genetic component of SLE is especially rich in genetic factors with effects above the detectable level with current studies (OR = 1.15 to 1.25). We are therefore now in a phase of exciting discoveries in this field. There still remain formidable challenges, however, because it is necessary to transform the information we obtain into useful knowledge and, as has been discussed above, we have very few clues regarding the meaning of the identified SLE associations. Future studies should try to identify the causal variants and to determine their effect at molecular, cellular and disease levels, including the assessment of their role in the different SLE phenotypes and the probable similar effect in women and men.

## Abbreviations

BANK1: B-cell scaffold protein with ankyrin repeats 1; BLK: B-lymphoid tyrosine kinase; GWA: genome-wide association; IL: interleukin; ITGAM: integrin alpha M; LY9: lymphocyte antigen 9; MECP2: methyl CpG binding protein 2; OR: odds ratio; PXK: PX domain containing serine/threonine kinase; SLE: systemic lupus erythematosus; SLEGEN: International Consortium for Systemic Lupus Erythematosus Genetics; SNP: single nucleotide polymorphism; STAT4: signal transducer and activator of transcription; TYK2: tyrosine kinase 2.

## Competing interests

The authors declare that they have no competing interests.

## Authors' contributions

MS-G participated in design of the study, in genotyping the samples, in interpretation of the results and in writing the manuscript. MC participated in the statistical analysis and in the interpretation of results. EE, RP, JO-R, GDS, SR, MJS, CP, MM, FNS, AS, FJB, SD'A, MB, PC, TW and SM participated in the acquisition of clinical data and collection of samples and in the analysis and interpretation of results. JJG-R coordinated the acquisition of clinical data and collection of samples and participated in the analysis and interpretation of results. AG participated in the design of the study and in the coordination of acquisition of clinical data and collection of samples, and supervised genotyping, statistical analysis, interpretation of results and writing of the manuscript. All authors read and approved the final manuscript.

## Authors' information

Other contributors to the European Consortium of SLE DNA Collections: Attila Kovacs (Albert Szent-Györgyi Medical and Pharmaceutical Centre, University of Szeged, Hungary); Rudolf Pullmann Jr (Gerontology Research Center, National Institute on Aging, Baltimore, MD, USA); Eva Balada (Hospital Vall d'Hebron, Barcelona, Spain); Ctibor Dostal (Institute of Rheumatology, Prague, Czech Republic); Filipe Vinagre (Hospital Garcia de Orta, Almada, Portugal and Instituto Medicina Molecular, Faculdade de Medicina da Universidade de Lisboa, Portugal); Iris Kappou-Rigatou (Evangelismos Hospital, Athens, Greece); Raffaella Scorza (University of Milan and Fondazione IRCCS Ospedale Maggiore Policlinico, Mangiagalli e Regina Elena, Milan, Italy); Maria Mavromati (Athens University Medical School, Athens, Greece); Carmen Gutierrez (Hospital Universitario Central de Asturias, Universidad de Oviedo, Spain); Ignacio Rego (INIBIC-CH Universitario A Coruña, Spain); Nadia Barizzone (Eastern Piedmont University, Novara, Italy); Cees G Kallenberg (University Medical Center Groningen, The Netherlands); and Reinhold E Schmidt (Hannover Medical School, Hannover, Germany).

## Note

A report published in *Arthritis & Rheumatism *after publication of this manuscript provided further confirmation of the association of *MECP2 *with SLE [26].

## Supplementary Material

Additional file 1 A Word file containing Table S1 that lists the origin and female percentage of the DNA sample collections, Table S2 that lists the clinical characteristics of the patients with SLE, Table S3 that lists the primers and probes used for genotyping the 10 SNPs, Table S4 that lists the genotype counts for each of the 10 SNPs detailed for each of the sample collections, and Table S5 that lists the minor allele percentages for each of the 10 SNPs for each of the sample collections. Click here for file
