# Supplementary material for: Replication of recently identified systemic lupus erythematosus genetic associations: a case–control study
Source: Arthritis Res Ther. 2009 May 14;11(3):R69. doi: 10.1186/ar2698 (PMC2714115; doi:10.1186/ar2698)
Supplement: Additional file 1 — A Word file containing Table S1 that lists the origin and female percentage of the DNA sample collections, Table S2 that lists the clinical characteristics of the patients with SLE, Table S3 that lists the primers and probes used for genotyping the 10 SNPs, Table S4 that lists the genotype counts for each of the 10 SNPs detailed for each of the sample collections, and Table S5 that lists the minor allele percentages for each of the 10 SNPs for each of the sample collections. [file ar2698-S1.doc]

| Supplementary Table 1 | |  |  |  |  |  |  |  |  |  |  |
| --- | --- | --- | --- | --- | --- | --- | --- | --- | --- | --- | --- |
| Origin and female percentage of the DNA sample collections used for replication of SLE associated SNPs. | | | | | | | | |  |  |  |
|  |  |  |  |  |  |  |  |  |  |  |  |
| Collections | DNA samples | |  | Female % | |  |  |  |  |  |  |
|  | Control | SLE |  | Control | SLE |  |  |  |  |  |  |
| Czech Republic^a^ | 99 | 101 |  | 32.3 | 85.1 |  | ^a^ Institute of Rheumatology, Prague | |  |  |  |
| Germany^b^ | 92 | 82 |  | 25 | 90.2 |  | ^b^ Hannover Medical School | |  |  |  |
| Greece 1^c^ | 99 | 97 |  | 66.7 | 86.6 |  | ^c^ Evangelismos Hospital. Athens | |  |  |  |
| Greece 2^d^ | 88 | 94 |  | 93.2 | 91.5 |  | ^d^ Athens University Medical School | |  |  |  |
| Hungary^e^ | 95 | 95 |  | 48.8 | 88.4 |  | ^e^ Albert Szent-Györgyi Medical and Pharmaceutical Centre. Szeged | | | |  |
| Italy, Milan^f^ | 106 | 128 |  | 42.5 | 86.7 |  | ^f^ University of Milan and Fondazione IRCCS Ospedale Maggiore Policlinico | | | |  |
| Italy, Naples^g^ | 108 | 81 |  | 100 | 79 |  | ^g^ Second University of Naples | |  |  |  |
| Italy, Rome^h^ | 102 | 84 |  | 55.9 | 83.3 |  | ^h^ Ospedale S. Camillo – Forlanini. Rome | |  |  |  |
| The Netherlands^i^ | 180 | 104 |  | 58.9 | 86.5 |  | ^i^ University Medical Center Groningen | |  |  |  |
| Portugal^j^ | 95 | 94 |  | 89.5 | 85.1 |  | ^j^ Hospital Garcia de Orta. Almada | |  |  |  |
| Slovakia^k^ | 93 | 94 |  | 93.5 | 91.5 |  | ^k^ Martin Faculty Hospital. Martin | |  |  |  |
| Spain, Asturias^l^ | 200 | 147 |  | 69 | 91.8 |  | ^l^ Hospital Universitario Central de Asturias | | |  |  |
| Spain, Barcelona^m^ | 96 | 89 |  | 52.1 | 91 |  | ^m^ Hospital Val d’Hebron. Barcelona | |  |  |  |
| Spain, Coruña^n^ | 81 | 88 |  | 71.6 | 92 |  | ^n^ CH Universitario Juan Canalejo. Coruña | | |  |  |
| Spain, Madrid^o^ | 98 | 92 |  | 64.3 | 91.3 |  | ^o^ Hospital 12 de Octubre. Madrid | |  |  |  |
| Spain, Santiago^p^ | 95 | 109 |  | 48.4 | 89 |  | ^p^ Hospital Clinico Universitario de Santiago | | |  |  |
| Total | 1726 | 1579 |  | 63.2 | 88.2 |  |  |  |  |  |  |
